# Screening of *TYR*, *OCA2*, *GPR143*, and *MC1R* in patients with congenital nystagmus, macular hypoplasia, and fundus hypopigmentation indicating albinism

**Published:** 2011-04-15

**Authors:** Markus N. Preising, Hedwig Forster, Miriam Gonser, Birgit Lorenz

**Affiliations:** 1 Department of Paediatric Ophthalmology, Strabismology, and Ophthalmogenetics, University Medical Centre, University of Regensburg, Regensburg, Germany; 2 Department of Ophthalmology, Justus-Liebig-University Giessen, Universitaetsklinikum Giessen and Marburg GmbH, Giessen Campus, Giessen, Germany

## Abstract

**Background:**

A broad spectrum of pigmentation of the skin and hair is found among patients diagnosed with ocular albinism (OA) and oculocutaneous albinism (OCA). Even though complexion is variable, three ocular features, i.e., hypopigmentation of the fundus, hypoplasia of the macula, and nystagmus, are classical pathological findings in these patients. We screened 172 index patients with a clinical diagnosis of OA or OCA based on the classical findings, to evaluate the frequency of sequence variants in tyrosinase (*TYR*), P-gene, P-protein (*OCA2*), and the G-protein-coupled receptor 143 gene, OA1 (*GPR143*). In addition, we investigated the association of sequence variants in the melanocortin receptor 1 gene (*MC1R*) and *OCA2*.

**Methods:**

Pigmentation of the hair, skin, iris, and fundus were included in the evaluation of OCA and OA. Male OA patients showing X-linked inheritance were screened for *GPR143*. Females showing OA without family history were regarded as representing autosomal recessive OA (OA3). Direct sequencing was applied to PCR products showing aberrant single-strand conformation polymorphism–banding patterns.

**Results:**

Fifty-seven male index patients were screened for OA. We identified 16 potentially pathogenic sequence variations in *GPR143* (10 novel) in 22 males. In *TYR*, we identified 23 (7 novel), and in *OCA2* 28 (11 novel) possibly pathogenic variants. Variants on both alleles were identified in *TYR* or *OCA2* in 29/79 OCA patients and 14/71 OA patients. Sequence changes in *TYR* were identified almost exclusively in OCA patients, while sequence changes in *OCA2* occurred in OCA and OA patients. *MC1R* sequencing was performed in 47 patients carrying mutations in *OCA2* and revealed *MC1R* mutations in 42 of them.

**Conclusions:**

*TYR* gene mutations have a more severe effect on pigmentation than mutations in *OCA2* and the *GPR143* gene. Nevertheless, mutations in these genes affect the development of visual function either directly or by interaction with other genes like *MC1R,* which can be deduced from a frequent association of *MC1R* variants with p.R305W or p.R419Q in *OCA2*.

## Introduction

Albinism is a recessively inherited disease of disturbed melanin synthesis and/or melanin distribution in the epidermis, scalp, uvea, and retinal pigment epithelium. Melanocytes are the site of melanin synthesis, where black-brown eumelanin (found in eumelanosomes) and red-brown pheomelanin (in pheomelanosomes) can be distinguished. Eumelanin and pheomelanin production shares a common pathway, with L-3,4-dihydroxyphenylalanine (L-DOPA) being a common precursor.

In albinism, melanocytes fail to synthesize or distribute melanins properly, which results in oculocutaneous albinism (OCA) presenting with absent or reduced tanning, white, blond, or red-blond scalp, blue irides, and hypopigmented fundi due to a lack of pigments in the skin, hair, and eyes. Autosomal recessive ocular albinism (OA) and X-linked OA present respectively with hypopigmented retinal pigment epithelium and iris pigment epithelial cells only [1]. Albinism is an obvious condition in dark-pigmented populations. However, light complexion, blond hair, and blue irides are features frequent among nonalbinotic individuals in a population like that of Northern Europe, thus compromising their significance when it comes to a diagnosis of albinism. The classical ocular signs of albinism (nystagmus, macular hypoplasia, and hypopigmentation of the fundus) are more reliable in identifying patients with albinism, as they are more specific.

Five genes are currently known to be involved in the etiology of various nonsyndromic forms of albinism. Tyrosinase (*TYR*; OMIM 606933) and tyrosinase-related protein (*TYRP1*; OMIM 115501) catalyze the initial steps to melanin production, while P-protein (*OCA2*; OMIM 611409) and the solute carrier 45 subunit A2 (*SLC45A2*; OMIM 606202) are transporters localized in the melanosome membrane. Variants in *TYR* [2], *OCA2* [3], *TYRP1* [4], and *SLC45A2* [5] underlie autosomal recessive OCA (OMIM 203200, 606952, 203100, 203290, 606574), and autosomal recessive OA (OA3) is caused by hypomorphic variants in *OCA2* [6]. Several reports of an association of *TYR* variants with OA3 [7-9] have appeared, but these findings were not confirmed in a recent study on the p.R402Q variant in *TYR* [10]. X-linked OA (OA1; OMIM 300500) is caused by variants in *GPR143*; OMIM 300808 [11], a transmembrane receptor in melanocytes of unknown function [11].

Eumelanosomes develop upon activation of the α-melanocyte-stimulating hormone receptor (*MC1R*; OMIM 155555) by α-melanocyte-stimulating hormone in response to ultraviolet light [12]. Mutant *MC1R* impairs eumelanosome development and supports pheomelanosome development, resulting in red hair and pale skin phenotypes [13,14].

The exact disease mechanism leading to nystagmus, macular hypoplasia, and optic nerve misrouting in albinism is still unknown. *TYR*, *OCA2*, and *SLC45A2* are involved in melanin production, but it is unknown how they contribute to the development of the retina and the visual system. Recent data indicate that L-DOPA may be a ligand for the protein encoded by *GPR143* [15]. L-DOPA is a precursor in melanin synthesis that has been considered as an antimitogenic factor in cell cycle regulation, playing a crucial role in the maturation of the retina and the optic nerve [16,17].

Recent results by King et al. [18] indicate that *MC1R* variants in combination with *OCA2* variants may lead to persistent red hair color (RHC) in OCA2 patients after birth. Interestingly, MC1R variants alone are insufficient to cause reduced visual functions in red-haired probands, as shown in many studies on the involvement of *MC1R* variants in RHC [19,20]. These observations may be taken to indicate that early products of the melanin pathway up to DOPA quinone underlie the abnormal neuronal development and reduced visual function in albinism. In this regard, it is unclear if it is a problem of production or distribution of neuroactive components.

In this study, we screened 172 patients with classical ocular signs of albinism (nystagmus, macular hypoplasia, and hypopigmentation of the fundus) for sequence variants in *TYR*, *OCA2*, *GPR143*, and *MC1R*. Here, we describe the prevalence of sequence variants of the analyzed genes in different albinism phenotypes and report a set of novel sequence variants. We also show that *OCA2* sequence variants p.R305W and p.R419Q are more often combined with sequence variants in *MC1R* than would be expected from the current literature.

## Methods

This study was approved by the ethical review boards of the Medical Center of the University of Regensburg and the Medical Faculty of the Justus-Liebig-University Giessen, Germany. Informed consent was obtained from all probands or parents according to the Declaration of Helsinki.

### DNA samples were collected between 1998 and 2008

One hundred and seventy-two index patients with nystagmus, hypopigmentation of the fundus, and macular hypoplasia were screened. One hundred and forty-two were of Caucasian origin from Germany or Austria. Ten patients were from Turkey, four from Italy, and single patients were from the Balkan States, Africa, Asia, and the USA. Seventy-nine index patients were diagnosed with OCA and 83 index patients with OA. Fifty-seven index patients with OA were male. Additional clinical data were available for 114 out of 172 patients (57 OCA, 57 OA).

Seventy-eight patients were referred for genetic analysis by external physicians and human geneticists. Ninety-four patients were examined by the authors at the Department of Paediatric Ophthalmology, Strabismology, and Ophthalmogenetics at the Medical Centre of the University of Regensburg (1998 to 7/2007) or the Department of Ophthalmology at the Universitaetsklinikum Giessen and Marburg GmbH, Giessen Campus (from 8/2007 onwards). The clinical examination of the patients included visual acuity, refraction, iris translucency, fundus appearance, and glare sensitivity. In selected patients, testing of the albinism-specific crossing of nerve fibers was performed by visually evoked potentials (VEP) [21]. Nystagmus, ocular hypopigmentation, macular hypoplasia, and pedigree information were used as criteria for further clinical examinations [22]. Differentiation between OA and OCA was attempted by recording skin tanning and hair color.

The mean refractive error was computed based on the spherical equivalent calculated from measurements of sphere and cylinder values (sphere - 0.5 cylinder=spherical equivalent). Subsequently, a mean value was computed for each group of patients (OCA, OA1, OA3).

DNA was extracted from peripheral blood lymphocytes and isolated according to Miller et al. [23]. The coding exons of *OCA2* (GenBank: NG_009846.1, NM_000275.2) and *GPR143* (GenBank: Z48804, NM_000273.2), including adjacent noncoding and flanking sequences, were amplified by PCR using oligonucleotides according to Lee et al. [24] and Schiaffino et al. [25]. Oligonucleotides for *TYR* (GenBank: M27160, NM_000372) were redesigned to improve access to single-strand conformation polymorphism (SSCP) analysis (Table 1). *MC1R* (GenBank: NG_012026) was amplified and directly sequenced using oligonucleotides designed as described in Table 1. Except for *MC1R,* oligonucleotides used for PCR were also used for sequencing.

**Table 1:** Sequences and amplification condition for primers designed by the authors.

**Name**	**Product**	**Sequence (5′-3′)**	**Annealing temperature**	**Product** **size**
**TYR Primers**				
Tyr-11f	Exon 1, 5′ part	CCAATTAGCCAGTTCCTGCAGA	60°, 4% DMSO	345 bp
Tyr-11r		CACAGTTGAATCCCATGAAGTTGC		
Tyr-12f	Exon 1, center	TATAATAGGACCTGCCAGTGCTCTG	61°, 4% DMSO	342 bp
Tyr-12r		AATGTCTCTCCAGATTTCAGATCCC		
Tyr-13f	Exon 1, 3′ part	TGTGTCAATGGATGCACTGCTT	60°, 4% DMSO	331 bp
Tyr-13r		AGAAGTGATTGTTAAGGTTCCTCCC		
Tyr-2f	Exon 2	TTGTTTAACATGAGGGTGTTTTGTACAG	60°, 4% DMSO	313 bp
Tyr-2r		GGACTTTGGATAAGAGACTGTAAATATG		
Tyr-3f	Exon 3	ATAATTATAAATCAATCACATAGGTTTTCA	55°	263 bp
Tyr-3r		CCAATGAGCACGTTATTTATAAAGA		
Tyr-4f	Exon 4	AAAATTTTCAAATGTTTCTTTTATACACA	56°, 4% DMSO	280 bp
Tyr-4r		CAGCAATTCCTCTGAAAGAAAGTAA		
Tyr-5f^a^	Exon 5	TGAAAGGATGAAGATGATGGTGATC	61°, 4% DMSO	350 bp
Tyr-5r^a^		TTGAGTTAGAGTGAGGTCAGGCTTTT		
**OCA2 Primers**				
PG2f	Exon 2	AGTGGTTTCTTTCTGGCTGCCC	60°	313 bp
PG2r		TGAAGTCCACATTTACAAGATGGCA		
PG251f	Exon 25	TCTCATGAGCTTATCCAGATTTCAGA	60°	222 bp
PG251r		GTGGGGTCAGGGTAGTTTTATGACTA		
**MC1R Primers**				
MC1Rf	5′ sense	ACTTAAAGCCGCGTGCACCG	65°, 4% DMSO	1016 bp
MC1Rr	3′ antisense	AGGCCTCCAACGACTCCTTCCT		
**Sequencing primers**				
MC1Ria	internal sense	GGTGCTGCAGCAGCTGGACAAT		
MC1Rib	internal antisense	AGAAGACCACGAGGCACAGCAGGAC		

All amplifications were run in a program of 94 °C denaturation for 5 min, 35 cycles of annealing (T_ann_ see in table), elongation (72 °C), and denaturation (94 °C) for 1 min and completed for a final annealing at 1 min and elongation for 10 min closing with 10 min at 10 °C. ^a^Oligonucleotides used to amplify exon 5 of TYR also amplify exon 5 of the TYR-pseudogene.

SSCP analysis was performed using nondenaturing PAGE (PAGE) [26] on Multigel-Long and Maxigel chambers (Biometra Whatman, Göttingen, Germany) at 10 °C. Two types of gels were applied: 8% polyacrylamide and 10% polyacrylamide in 1x TBE buffer. Ten percent polyacrylamide gels were overlaid with a 6% polyacrylamide gel in 1x TBE. Both types of polyacrylamide gels were used with and without a glycerin content of 5%. PCR products showing aberrantly migrating bands in SSCP were submitted to direct sequencing (Seqlab, Göttingen, Germany).

The sequence changes found were confirmed by restriction enzyme digestion whenever an RFLP was created, or by noncoding strand sequencing.

If available, both parents and additional family members were tested for the identified variants in *TYR* and *OCA2,* either by direct sequencing or restriction enzyme digestion. Mothers and relatives at risk of males presenting with *GPR143* variants were tested for their carrier state. *MC1R* variants were tested in available relatives of patients with identified *OCA2* sequence variants to evaluate their association with *MC1R* variants.

Missense variants were assessed on the PolyPhen server (June 2009) and SIFT server (June 2009). Splice site and intronic variants were assessed using the Human Splicing Finder server (June 2009). The Predict Protein Server (June 2009) and the PHYRE server (July 2009) were used for secondary structure prediction to evaluate the functional impact of some *OCA2* variants.

## Results

### Patients and clinical data

Hypopigmentation of the fundus was seen in all patients. The extent of fundus hypopigmentation went along with iris translucency findings. Findings for iris translucency were obtained in 114 patients. Mild iris translucency with only a few widely distributed spots was seen in 19/41 of the patients without the identification of an underlying variant in the genes screened. No or only light iris translucency was described in 50% of OCA patients, while obvious or full iris translucency was seen in 25% of patients diagnosed with the OA1 or OA3 sequence variant. A general correlation between positive variant identification and the intensity of iris translucency could not be found.

**Figure 1:**
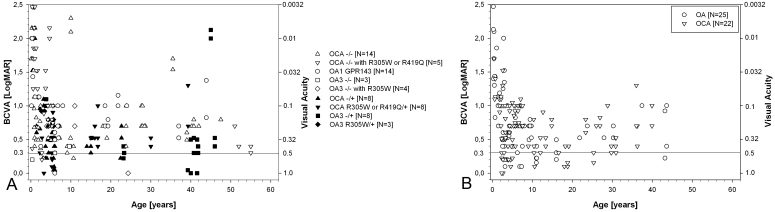
Summary of best corrected visaul acuity (BCVA) data obtained in oculocutaneous albinism (OCA) and ocular albinism (OA) patients. **A**: Open symbols denote patients with identified sequence changes on both alleles; closed symbols denote patients with identified sequence changes on a single allele only. **B**: Patients without identified variants. Visual acuity is given as the negative logarithm of the minimum angle of resolution (logMAR) and was obtained with Teller acuity cards (6 months-3 years), Cardiff Crowding cards (up to 3 year of age), Lea cards (2–6 years), and number charts (6 years and older). Single patients are presented with follow-up data. Nonquantifiable data were transferred into digital data according to Schulze-Bonsel and Bach (hand movement: logMAR 2.5, counting fingers: logMAR 2 [42]).

**Figure 2:**
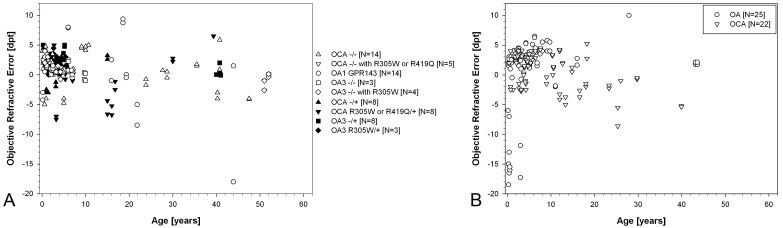
Summary of objective refractive error (including normalized spherical error) obtained by retinoscopy in oculocutaneous albinism (OCA) and ocular albinism (OA) patients. **A**: Open symbols denote patients with identified sequence changes on both alleles; closed symbols denote patients with identified sequence changes on a single allele only. **B**: Patients without identified variants. Single patients are presented with follow-up data.

Visual acuity was measured at logMAR>2.0 in the first months of age and at logMAR 0.22 in childhood (mean 0.6, Figure 1). Refraction was generally hypermetropic. OCA patients with identified variants (29 patients) presented with a mean refractive error of +1 D (range −7.5 D to 10 D), OA1 patients (14 patients) with a mean refractive error of +0.6 D (range −18 D to +9.4 D), and patients with OA3 (18 patients) with a mean refractive error of +1.9 D (range −1 to +2.5 D, Figure 2).

Comprehensive data for skin, hair, and eye color were available for 21 out of 106 patients with a positive variant detection in at least one allele of *TYR*, *OCA2*, or *GPR143*.

### Sequence variants in X-linked ocular albinism (OA1)

Screening for sequence variants in *GPR143* was performed in 57 male patients with OA (Table 2). Ten of the patients had a positive family history for X-linked inheritance. Twenty-two male OA patients (39%) carried at least one of 15 nonsynonymous variants in *GPR143*, including eight novel variants (Appendix 1). The prevalence of *GPR143* variants increased to full coverage when X-linked inheritance could be ascertained. Six of the 15 identified *GPR143* variants were missense variants. A second variant (p.I276V) occurred on the same allele in 2 out of 5 index patients with p.G312V. The remaining nine variants in *GPR143* caused premature stop codons either by nonsense variants (2 patients) or frameshifting variants (7 patients, Appendix 1 and Appendix 2).

**Table 2:** Overview of the 172 index cases screened and the number of identified variants.

**Patients identified^c^**	**Index cases**	**None**	**Autosomal**				**X-linked GPR143**	
			**TYR**		**OCA2**			
			**Single**	**Two**	**Single**	**Two**	**None**	**Hemizygous**
OCA	79	27 (35)	6 (8)	9 (11)	17 (21)	20 (25)		
OA female	36	19 (53)	4 (11)	2 (6)	6 (17)	5 (14)		
OA male^a^	57/10^b^	18 (32)	2 (4)		8 (14)	7 (12)		22 (39)/10^b^

^a^males with a diagnosis of OA, ^b^males with an obvious X-linked inheritance, ^c^numbers indicate individuals, numbers in parentheses indicate percentages.

### Sequence variants in autosomal recessive oculocutaneous albinism

Seventy-nine patients diagnosed with OCA were screened for variants in *TYR* and *OCA2*. In these patients, we identified 23 nonsynonymous variants in *TYR* (7 novel, Appendix 1), and 28 variants in *OCA2* (11 novel, Appendix 1). *TYR* variants on both alleles were identified in nine patients (11%) with OCA (Table 2 and Appendix 2). Single heterozygous conditions in *TYR* and *OCA2* were identified in 23 patients (Appendix 1 and Appendix 2) with OCA (30%). The majority of *TYR* and *OCA2* sequence changes altering the amino acid sequence in this study were missense variants (83% in *TYR* and 79% in *OCA2*). Four variants in *TYR* and *OCA2* each predicted a preterm translation stop; 2 of 7 novel missense variants in *TYR* were predicted to be nonpathogenic (Appendix 1).

*OCA2* variants on both alleles were identified in 20 OCA index patients (25%, Appendix 2). The variant spectrum in *OCA2* was broader: Twenty-three of the 28 sequence variations predicting changes in the primary structure of the protein were missense variants. The remaining sequence changes predicted a preterm translation stop by a nonsense variant, a single nucleotide deletion inducing a frameshift, or splice site variants. Two of 11 novel variants in *OCA2* were rated nonpathogenic (Appendix 1). Single heterozygous sequence variations were identified in *TYR* in six patients (8%), and in *OCA2* in 17 patients (21%).

### Sequence variants in autosomal recessive ocular albinism (OA, OA3)

Thirty-five male OA patients and 36 female OA patients were screened for variants in *OCA2* and *TYR*. Seven of the male OA patients (12% of all OA males screened) showed compound heterozygous variants in *OCA2*; none were identified in *TYR*. Among female patients showing OA3 and screened for *TYR* and *OCA2* variants, we identified seven (20%) sequence variants in *TYR* (2 variants) and *OCA2* (5 variants).

p.R402Q was found in the homozygous state twice and in nine compound heterozygous combinations with other *TYR* gene variants (9.9% in this study, Appendix 2). In our cohort, p.R402Q was equally frequent identified in patients with the OCA phenotype (8 patients) versus OA (10 patients).

### Evaluation of sequence variants in *MC1R*

*MC1R* variants were identified in 13 of 18 index patients (72%) carrying *OCA2* variants p.R305W or p.R419Q in the single heterozygous state, and in 12 of 17 index patients (71%) carrying *OCA2* variations p.R305W or p.R419Q as one of the compound heterozygous alleles. The *MC1R* sequence changes included strong RHC alleles (p.R151C, p.I155T, p.R160W, p.D294H) and weak RHC alleles (p.V60L, p.V92M, p.R163Q) of *MC1R* (Appendix 1). Testing the available parents for single heterozygous *OCA2* variants revealed one unaffected mother (271.2) cosegregating *OCA2*^R305W^ and *MC1R*^V60L^.

## Discussion

We screened 172 patients with clinical signs of albinism for sequence variations in genes most prevalent in human OCA and OA (*TYR*, *OCA2*, and *GPR143*). Congenital nystagmus was the common symptom in all patients, accompanied by fundus hypopigmentation, macular dysplasia, and iris translucency of varying degree.

### Sequence variants of *GPR143* in X-linked ocular albinism

Variants were identified in only 22 of 57 male patients screened for *GPR143,* but were represented in 100% of patients with an obvious family history of X-linked inheritance. This observation underlines the importance of a formal genetic workup before molecular genetic analysis. Eight of 35 male patients in which *GPR143* variants were excluded by screening were found to carry pathogenic sequence changes in *OCA2*; these results are discussed below.

As we reported previously [26], nystagmus and macular hypoplasia are the prominent symptoms of OA1 [27]. Therefore, recent reports of *GPR143* underlying isolated X-linked congenital nystagmus (NYS6; OMIM 300814) [28-30] should be considered with caution. Other ocular features, though present, may be less prominent, as shown by Liu et al. [30] and our previous study [26], in patients showing stronger pigmentation. The reports by Peng et al. [28] and Zhou et al. [29] did not present sufficient clinical data to evaluate their hypothesis of isolated nystagmus from *GPR143* variants. Therefore, male patients with congenital nystagmus are candidates for X-linked OA and need a thorough clinical examination for this condition.

### Sequence variants in *TYR*

The prevalence of disease causing *TYR* sequence variants on OCA was 11% in this study. This is explained by the predominant diagnostic criteria used for the oculocutaneous patient cohort in this study, leading to a bias toward the OCA2 phenotype. Other studies showed comparable prevalences [31]. The present study also supports the notion that *TYR* is not frequently involved in OA3 because only 3% of our cohort of OA3 patients presented with pathogenic sequence variants in this gene. In the present study, p.R402Q was predominantly identified in patients showing the OCA phenotype. This observation contrasts with of those of Hutton and Spritz [9], who identified compound heterozygous combinations with p.R402Q predominantly in OA3. Our findings are comparable with data by Grønskov et al. [32], which showed that *TYR* variants are rare in OA3 patients.

In addition, Oetting et al. [10] identified p.R402Q in the compound heterozygous state with other pathologic variants in unaffected relatives of OA3 patients. We therefore analyzed p.R402Q in unaffected relatives of our patients and identified one unaffected parent carrying p.R402Q in the homozygous state (286.3), thus arguing against a pathogenic effect.

### Sequence variants in *OCA2*

Pathogenic sequence variants in *OCA2* were more frequent in our patient cohort than *TYR* variants (25% versus 11% in OCA, 17% versus 3% in OA3). These findings suggest that mutations in additional genes remain to be identified in patients fulfilling the selection criteria that were applied in this study.

Two splice site variants were identified in *OCA2* that have been known for a long time (Appendix 1). c.1113T>C has been regarded as an isocoding polymorphism (p.G371) at the amino acid level. An evaluation of this variant on the Splice Sequence Finder Server has now demonstrated the gain of a novel splice donor site, which has not been considered before. We identified this variant in two patients, one in the homozygous, and one in the compound heterozygous state, supporting its pathogenicity. Patient 979.1 presented with OCA and was of Turkish origin. The patient carried c.1113T>C in the compound heterozygous state with p.R419Q in *OCA2* and p.R160W in *MC1R*. A Vietnamese OCA patient (2117.2) showed the c.1113T>C variant in the homozygous state. In addition, he carried a second homozygous variant in *OCA2* (p.G775S [c.2323G>A]). Evaluation by PolyPhen and SIFT were contradictory; therefore, p.G775S could not be rated pathogenic or nonpathogenic. This subject’s affected mother (2117.1) and father (2117.3) both carried the same combined allele ([c.1113C>T, p.G775S]) heterozygously. The affected mother carried an additional common variant of the Asian population (p.H615R [c.1844A>G]), supporting our interpretation [33].

Six of the nonsynonymous sequence variations in *OCA2* were consistently rated as benign by PolyPhen and rated as tolerated by SIFT. Interestingly, three of the variants classified as benign were previously accepted as pathogenic *OCA2* variants, including p.V443I, a frequent variant in Caucasians, and p.A481T, a frequent OCA variant in Japanese [34] (Appendix 1). This finding highlights the limitations in predicting the functional consequences of *OCA2* variations, which is likely due to the lack of data on their secondary and tertiary structure. The functional and structural prediction of the N-terminal 320 amino acids is insufficient, since only vertebrate sequence data are available for *OCA2* in the Entrez Database.

Other frequently encountered sequence variations in *OCA2* included p.D257A, p.R305W, and p.R419Q (Appendix 1). p.D257A was identified in the homozygous state in three unaffected relatives in our study, thus arguing against a functional impact.

Evaluations made by PolyPhen and SIFT predicted p.R305W as deleterious or not tolerated (see Appendix 1). Alignments in the Vector NTI 11 suite (AlignX, Invitrogen, Karlsruhe. Germany) using sequence data from various vertebrate species obtained from the Entrez Database showed glutamine to be the predominant amino acid at this position. A single deviation from the rule (arginine) was present in the *S. scrofa* sequence only. Tryptophan was never present at this position in the sequence data screened. Structural prediction by the PredictProtein Server indicated a decrease in the globularity of the protein by p.R305W. This affects the stability of the β-sheet structure where p.R305 is located.

p.R419Q is positioned within a loop region in the anterior permease domain of the P-protein. The variant is predicted to increase the globularity of the P-protein without any predicted effect on the β-sheet or α-helix formation in this region (PredictProtein Server). The amino acid position is highly conserved among vertebrates.

Several studies have evaluated the influence of p.R305W and p.R419Q on pigmentation [24,35-39]. These studies excluded an influence of both variants on hair color but supported an effect on skin color, since the patients predominantly showed brown hair and light skin complexion.

The reported prevalence of p.R305W in the Northern European population is given at 5%, while p.R419Q is reported at 9% [37]. In this study, we identified p.R305W in 8.4% and p.R419Q in 4.7% of all alleles typed for *OCA2* (Appendix 1). p.R305W was predominantly identified in index cases of OCA (9 patients with p.R305W versus 4 patients with p.R419Q of 21 patients carrying two mutant *OCA2* alleles) and p.R419Q was predominantly identified in OA3 index cases (4 patients with p.R419Q versus 2 patients with p.R305W of 14 patients carrying two mutant *OCA2* alleles). This indicates a yet-unidentified effect of p.R305W and p.R419Q in patients presenting with nystagmus, macular hypoplasia, fundus hypopigmentation, and iris translucency. Pigmentation of the skin and hair used to classify OCA from OA may therefore depend on the compounding variants and possibly on other genes associated with pigmentation.

### Influence of *MC1R* variants

King et al. [18] already reported on p.V443I and other *OCA2* variants associated with *MC1R* variants modifying the *OCA2* phenotype. We tested the hypothesis of the association of *MC1R* variants with *OCA2* variants in this study. Combinations of p.V443I and MC1R variants were identified in two patients (810.2 and 1128.1). In addition, we observed a very frequent association of p.R305 W and p.R419Q alleles in *OCA2* with sequence changes in *MC1R* in this study. Presumably, p.R305W and p.R419Q were not considered in previous reports because they were regarded as polymorphisms. A specific disease-related interaction with *MC1R* has not been excluded so far.

Kanetsky et al. [40] reported *MC1R* variants in 46.7% of the alleles tested in nonalbino US Caucasians. These probands frequently showed light pigmentation phenotypes in eyes, hair, and skin in double heterozygous as well as single heterozygous patients. A liberal estimation of the allele frequency for any *OCA2* sequence variations in albinism patients at about 25% is supported by the literature [32]. Therefore, the coincidence of *OCA2* variants and *MC1R* variants should not exceed 12%–13%, since they are independently inherited. This is far below the frequency of 72% of index patients carrying MC1R variants associated with p.R305W and p.R419Q in a biallelic condition in this study.

All variants in *MC1R* cosegregating with *OCA2^R305W^* or *OCA2^R419Q^* despite *MC1R*^V60L^ were predicted to reside on the cytoplasmic side at the intersections to and from transmembrane domains allowing interaction with the P-protein. MC1R^V60L^ is located on the outside surface of the protein, which limits its potential to interact with the P-protein.

Therefore, we consider p.R305W and p.R419Q to be disease causing alleles, and assume an effect on interaction with *MC1R* that leads to visual dysfunction. *MC1R* provides the basis for reduced pigmentation, and P-protein that interacts with MC1R may influence cellular functions necessary for the secretion of growth factors during neural development. Probably, the interaction between MC1R and P-protein does not affect the production of melanin itself, but rather the distribution or production of a precursor like L-DOPA. In this scenario, a minor effect on pigmentation would result in a broad spectrum of pigmentation phenotypes and a low number of patients with reduced visual function and light complexion. Further functional studies will help to clarify the situation.

## Appendix 1. Evaluation of the effect of variants identified in this study.

To access the table, click or select the words “Appendix 1.” This will initiate the download of a pdf archive that contains the table. ^a^84 index alleles in analysis, ^b^332 index alleles in analysis, ^c^344 index alleles in analysis, ^d^in cis with p.G312V, ^e^114 index alleles in analysis, ^f^prediction was performed by 1. PolyPhen, psd: possibly deleterious, pbd: probably deleterious, b: benigne, 2. SIFT, nt: not tolerated, tol: tolerated, 3: SSF, IVS: Intron (Intervening sequence), gray shaded rows indicate variants within tyrosinase copper binding domains, GenBank accession numbers: GPR143 (Z48804, NM_000273.2), OCA2 (NG_009846.1, NM_000275.2), TYR (M27160, NM_000372.4), MC1R (NG_012026); RISN – Variant was reported previously and is listed in locus specific database at the Retina International Scientific Newsletter ^g^yes: segregates with the phenotype inside the family, no: does not segregate, unknown: no further affected relative available for testing, RHC: Raid Hair Color Allele [19].

## Appendix 2. Index case related summary of the genetic data obtained in this study

To access the table, click or select the words “Appendix 2.” This will initiate the download of a pdf archive that contains the table. ^a^Shared cells indicate alleles that could not be tested for segregation, con: consanguineous, ex: ecluded, nd: not done, na: not available, Variant identified in M mother, F father, D daughter, S sister, B brother, CM male cousin, U uncle, A aunt, GF grandfather, GM grandmother, ^b^Country codes: A: Austria, D: Germany, E: Spain, HR: Croatia, I: Italy, SRB: Serbia, TR: Turkey, VN: Vietnam, NL: The Netherlands, ^c^Variant was reported by King et al. [16] with a comparable phenotype. ^d^Patients were reported by Preising et al. [26]. ^e^Patients were reported by Preising et al. [41], Squared brackets indicate variants residing on the same allele. *MC1R* could not be tested in patient 627.1 due to lack of DNA. The parental DNA was analyzed instead and revealed two variants in each parent shown in the table. We could not show the segregation of these variants, so we placed the variants in parentheses to indicate possible co-segregation.
